# Screening for Nephropathy in Diabetes Mellitus: Is Micral-Test Valid among All Diabetics?

**DOI:** 10.1155/2016/2910627

**Published:** 2016-05-18

**Authors:** Koubaa Afifa, Sriha Belguith Asma, Harzallah Nabil, Bellaleh Ahlem, Sahtout Mounira, Younes Kawthar, Triki Sonia, Hellara Ilhem, Neffati Fadoua, Najjar Fadhel, Soltani Mohamed

**Affiliations:** ^1^Stah Jabeur Primary Health Center, Monastir Health Group, 5000 Monastir, Tunisia; ^2^Department of Preventive Medicine and Epidemiology, Faculty of Medicine, Monastir, Tunisia; ^3^Primary Health Center, Monastir Health Group, Monastir, Tunisia; ^4^Department of Pathology, Monastir, Tunisia; ^5^C2 Primary Health Center, Monastir Health Group, Monastir, Tunisia; ^6^Biochemistry Laboratory, Faculty of Pharmacy, Monastir, Tunisia

## Abstract

*Background. *Using Micral-test (MT) for screening microalbuminuria (MA) among type 2 diabetics (T2D) is helpful. We aimed at determining prevalence of MA and at describing the MT validity.* Methods.* We studied 182 T2D followed up in family medicine. Two 24-hour urinary quantitative assays of MA had been used as a gold standard.* Results*. Prevalence of MA was 23%, CI 95%: 16.9–29.1. MT validity was 77% for sensitivity, 88% for negative predictive value, and 0.2 for Kappa coefficient (*p* = 0.001). Among subjects having a blood pressure ≥130/80 mmHg, having a CHT/HDL ratio ≥ 3, being a T2D for more than 5 years, and being women, negative predictive values were, respectively, 91%, 89%, 95%, and 91%. The area under the ROC curve was 0.81 in men (*p* = 0.008) and 0.80 when diabetes duration exceeds 5 years (*p* = 0.001). The MA value at 100% Sp for MT was 35 mg/L.* Conclusion.* The use of MT in primary healthcare for yearly screening for MA in T2D must be accentuated especially when diabetes duration exceeds 5 years or when associated with other cardiovascular risks.

## 1. Introduction

Screening and management of early stage of diabetes are insured by the family practitioner, while Tunisian diabetics with complications are managed by specialists. Diabetic nephropathy (DN) is the commonest cause of end-stage renal disease (ESRD) [1]. Screening for microalbuminuria (MA) is required in primary healthcare centers to prevent the progression of this serious complication [2–5]. The prevalence of DN is increasing along with the diabetes prevalence [6]. It should be detected and treated by the family doctor, at the stage of MA which is potentially reversible and associated with an increased risk of cardiovascular morbidity and mortality [7, 8]. The determination of MA by the semiquantitative method, Micral-test (MT), is a rapid screening tool. It is the easiest method, using urinary strips and a spot urine specimen. It is simple and inexpensive [9, 10]. The aim of this study was to assess the validity of MT criteria to detect DN among subgroups of diabetics and therefore to establish prevalence of MIA in diabetics managed in primary healthcare centers.

## 2. Methods

### 2.1. Design

This is a cross-sectional study, conducted from January 2013 to December 2014, to screen diabetic patients for elevated urinary albumin excretion with Micral-test in general practice.

### 2.2. Patients

We have included type 2 diabetics (T2D) without known complications followed up in two primary healthcare centers based in Monastir (“Stah Jabeur” and “C2”). Patients with fever, transitory diabetes decompensation, urinary infection, hematuria, or DN managed by a specialist were excluded from the study.

### 2.3. Study Area

Monastir is a coastal city located in the central eastern part of Tunisia. It includes 7 primary healthcare centers. Over than 20,000 inhabitants are followed up in “Stah Jabeur” and “C2” centers. Those centers were chosen because they manage diabetics at early stage.

### 2.4. Methods

The participants benefited from screening using the MT, done at the primary healthcare center. For the gold standard, two quantitative assays of MA on the urine of 24 hours were performed at one-month interval, at the Biochemistry Laboratory of the University Hospital of Monastir. MA was considered pathological if urinary albumin concentration (UAC) was between 30 and 300 mg in the urine of 24 hours. A third quantitative assay was performed in case of discordance. The MT is a semiquantitative strip test done on a urine sample in order to determinate MA. A comparative color scale is used to assess the results of MT: at a concentration of 0 mg/L of microalbumin in the urine, the strip remains white; it turns to light pink at 20 mg/L, to dark pink at 50 mg/L, and finally to very dark pink at more than 100 mg/L. A therapeutic education session was conducted on the test's day in primary healthcare center to improve patients' participation to MT and quantitative assays. Answerable nurse received information about this study.

### 2.5. Parameters

The participants also benefited from multiple blood tests. The studied parameters were sociodemographic data (age, gender), information about diabetes (diabetes duration, fasting blood glucose levels, glycated hemoglobin (HbA1C)), and risk factors associated with T2D (blood pressure, total cholesterol, and HDL cholesterol (HDL-C)). Total cholesterol to HDL-C ratio was calculated; the patient was considered as being at low risk of overload if this ratio was less than 3, at intermediate risk between 3 and 4.4, and at high risk if more than 4.5 [11]. Controlled glycemia is defined as the ADA [12]. Renal function was assessed by the measurement of glomerular filtration rate (GFR) calculated using the MDRD formula (modification of diet and renal disease). Some variables influence AUC, as was found in the literature. So we have analyzed validity criteria of MT according to demographic variables, blood pressure, CH/HDL-C ratio, and T2D duration.

### 2.6. Statistical Analysis

Statistical data were performed on SPSS 17.0 computer software. Sensitivity and specificity were calculated to determine the diagnostic properties of MT in predicting a UAC greater than or equal to 30 mg/24 hours. To determine the confidence intervals for sensitivity and specificity, we used the guidelines of Gardner and Altman. The coefficient Kappa (*κ*) was used to assess the concordance between the MT and the quantitative measurement of MIA. A value of *p* < 0.05 was considered as the cut-off of significance. The cut-off point for 100% specificity and the equilibrium point between sensibility and specificity were determined. We assessed, according to the subgroups, diagnostic sensitivity, specificity, positive and negative predictive values, Kappa coefficient, positive likelihood ratio, and negative likelihood ratio. The ROC curves of MT in diurnal random urine specimen for screening of MA according to the subgroups were plotted.

## 3. Results 

### 3.1. Description of the Study's Population

We have included 182 T2D among whom 122 were followed up in the primary healthcare center “Stah Jabeur” and 60 in “C2” center. The mean age of the patients was 61.4 years (SD: 11.9). Females represented 69.2% of the studied population (sex ratio = 0.44) and 62% had hypertension. The mean duration of diabetes was 8.1 years (SD: 6.4). Fasting blood glucose levels value was <7 mmol/L in 31% of the cases and the HbA1C <7% in 30% of T2D. Blood pressure was ≥130/80 mmHg in 66% of the studied population and 49% of the patients were obese. Mild renal impairment was present in 47.6% of diabetics. The prevalence of MA was 23%; CI 95% = 16.9–29.1.

### 3.2. Variability of Micral-Test Validity

**Figure 1:**
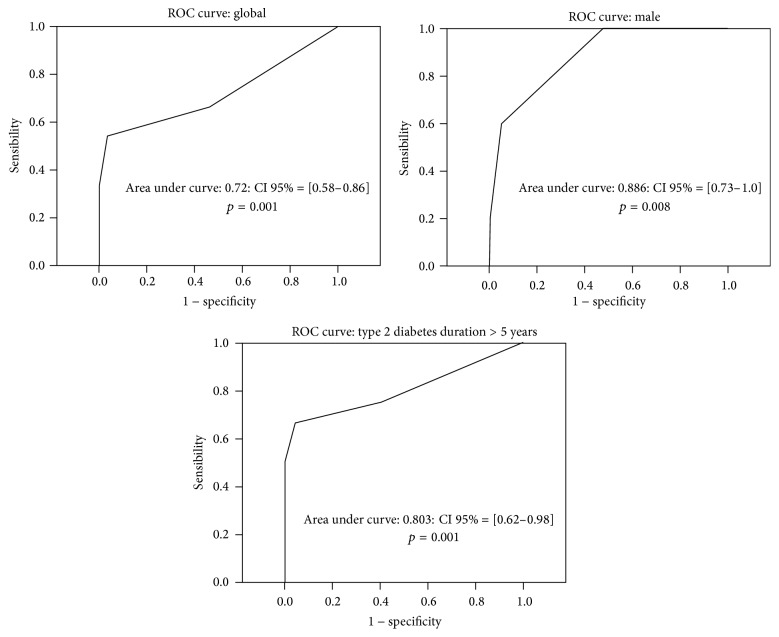
The area under the ROC curve of Micral-test (mg/L) in microalbuminuria screening.

The MT had 77% sensitivity, CI 95%: 65.5–88.9, 46% specificity, and a negative predictive value (NPV) of 88%. The positive likelihood ratio was 1.4 and the negative likelihood ratio was 0.5. The validity of MT was better in women (NPV = 91%; *κ* = 0.3; *p* = 0.008), subjects having a blood pressure ≥130/80 mmHg (NPV = 91%; *κ* = 0.24; *p* = 0.005) or a total CHT/HDL ratio ≥3 (NPV = 89%; *κ* = 0.36; *p* = 0.01), and patients who have had T2D for more than 5 years (NPV = 95%; *κ* = 0.38; *p* = 0.001) (Table 1). The cut-off point for 100% specificity was 35 mg/L for all studied subgroups. The equilibrium point between sensitivity and specificity was 10 mg/L (Table 2). The area under the ROC curves was 0.72: CI 95% = 0.581–0.863 (*p* = 0.001) for the entire study population. It was 0.81 in men (*p* = 0.008) and 0.80 when diabetes duration exceeds 5 years (*p* = 0.001) (Figure 1).

## 4. Discussion

The highlight of this study is that it was conducted in first-line therapy for diabetes in early stage. It allowed us to determine the accuracy of the MT in the detection of MA among diabetics subgroups and to estimate MA prevalence. This subject was little discussed in the Tunisian studies contrary to world literature [9, 10, 13–17].

**Figure 2:**
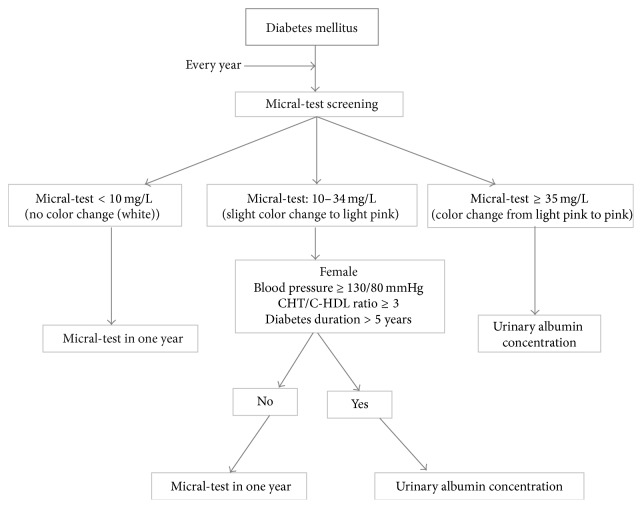
Algorithm for screening nephropathy in diabetes mellitus with Micral-test.

Our diabetic population was characterized by a female majority, as described in the literature [18, 19]. Different distributions were found though [20, 21]. The mean age (61.4 years) and the mean duration of diabetes (8.1 years (SD: 6.4)) were similar to those reported by other authors [13, 22]. We found that 30% of diabetics had a good glycemic control, and this result matches those observed in other Arab countries (Morocco, Kuwait, and Emirates) [13, 23, 24]. The glycemic control was better in China [25]. The review of literature shows a great variability in the prevalence of MA in T2D (Table 3); this prevalence ranged from 11.5 to 58.2% [13, 26]. This variation can be mainly attributed to the genetic and the ethnic dissimilarities between the studied groups [27] and to the difference in the classifications system. Among the studied population, 36 patients had MA (23%). This finding is comparable to those obtained in the United Kingdom Prospective Diabetes Study (UKPDS) [28] and in Adler et al.'s study [28] and Arfa et al.'s work [29] done in Tunisian specialized departments. MA's prevalence was lower in an Argentinean study [25] and greater in Canadian and Moroccan studies [13, 30, 31]. Several methods were used for the detection of the MA. The quantitative methods include the determination of the urinary albumin concentration (UAC) and the urinary albumin-to-creatinine ratio (UACR) in a first morning urine sample, 24 h urine collection, or timed urine collection. These two methods are performed in the laboratory by radioimmunoassay, radial immunodiffusion, enzyme immunoassay (ELISA), or immunoturbidimetric means. Among semiquantitative methods, the MT has been the most used screening tool for the detection of MA in many countries [32]. This test involves the immunochromatographic principle. Initially, the labeled antibody is combined with the sample albumin. Then, during the migration, excess conjugate is retained by the immobilized human albumin. Finally, the complex spread by migration on the strip to a revelation area. A more or less intense pink color appears in the play area based on the initial concentration of albumin [33, 34]. In different studies (Table 4), the MT precision varied from 67 to 96.7% and the specificity ranged from 46 to 96% [9, 32, 33, 35–39].  Table 5 shows a comparison between Micral-test and Multistix® commonly used for detection of DN in Tunisia. Our results are similar to Incerti's findings [36] for the same cut-off (20 mg/L). The MT validity was better in men (*p* = 0.008), subjects having a BP ≥130/80 mmHg (*p* = 0.005) or a cholesterol/C-HDL ratio ≥3 (*p* = 0.011), and patients who have had T2D for more than 5 years (*p* = 0.001). The point for 100% specificity (0% false positives) was 35 mg/L among all subgroups. The equilibrium point between sensibility and specificity was 10 mg/L; it was between 26 and 32 mg/L in Incerti et al.'s study [36]. Several studies have shown that the MT is commonly used worldwide, that it is a good screening tool for MA [32, 33, 36, 37, 39], and that positive results should be confirmed and quantified by a reference assay [33, 36]. Few authors have discussed the limitation of the diagnosis value of MT due to the rate of false positive [40], high costs, and the possibility of false negative results [32, 34]. Taking into consideration accuracy and cost, the measurement of the UAC in a random urine specimen, having 15 mg/L as the cut-off point for diagnosis, or the UACR on first morning micturition was considered by other authors as the best choice for MA screening patients with diabetes [36, 41]. Several advantages of MT have been described such as the economic benefit [42], the rapidity of the test (total time of 3 minutes), and its validity and reliability as a screening method for albuminuria on a single urine sample. It does not require 24-hour urine collection, feasible on a morning urine sample and especially adequate in the elderly [9, 32, 33]. We propose the algorithm (Figure 2) for screening ND.

## 5. Conclusion

Detecting MA in diabetic patients leads the family doctor to speed up the adaptation of the therapeutic goals in order to have the finest metabolic control, an improvement of health-related behaviors, and an optimal treatment of the associated risk factors. Screening for MA has to be improved in primary healthcare centers. The use of reliable, cheap, and easily semiquantitative methods such as the MT seems to be a promising strategy to achieve that purpose.

## Figures and Tables

**Table 1:** Assessment of validity of Micral-test according to subgroups of diabetics.

Cut-off value: 20 mg/L	Sensibility (%)	Specificity (%)	Kappa	*p*	NPV	PLR	NLR
*Entire population *	77.2 *CI 95%* [65.5–88.9]	46.3 *CI 95%* [32.4–60.2]	0.2	0.001	88.4	27.8	
*Gender*:							
(i) Male	66.6%	46.6%	0.103	0.008	77.7%	1.2	0.74
(ii) Female	77%	66%	**0.323**	0.006	**90.6%**	**2.26**	0.34
*BP ≥ 130/80 mmHg*	81%	54.1%	0.236	0.005	**90.7%**	1.76	0.35
*CHT/C-HDL ratio ≥ 3*	83.3%	60.1%	**0.363**	0.011	89.4%	2.07	**0.28**
*T2D duration > 5 years*	91%	60.6%	**0.378**	0.001	**95.2%**	**2.27**	**0.15**

NPV: negative predictive value.

PLR: positive likelihood ratio.

NLR: negative likelihood ratio.

**Table 2:** Sensitivity and specificity among subgroups depending on the chosen point.

	Cut-off point for 100% specificity for MT	Equilibrium point between sensibility and specificity
*Entire population *	*35 mg/L*	*10 mg/L*
Sensitivity	54.2%	66.7%
Specificity	100%	53.6%
*Gender*: *male*	35 mg/L	10 mg/L
Sensitivity	60%	**100%**
Specificity	100%	52.4%
*BP ≥ 130/80 mmHg*	35 mg/L	10 mg/L
Sensitivity	52.4%	66.7%
Specificity	100%	53.6%
*CHT/C-HDL ratio ≥ 3*	35 mg/L	10 mg/L
Sensitivity	58.3%	58.3%
Specificity	100%	57.5%
*T2D duration >5 years*	35 mg/L	10 mg/L
Sensitivity	**66.7**%	75%
Specificity	100%	60%

**Table 3:** Microalbuminuria's prevalence in the literature.

Study	Number of patients	MA measurement method	MA's prevalence (%)
Our study	182	24-hour urinary UAC: 30–300 mg/d	23%
Kuwait, 2008 [23]	440	Micral-test	14.2%
Argentina, 2011 [26]	88	24-hour urinary UAC: 30–300 mg/d	46.2%
Albania, 2013 [43]	222	Matinal urine UACR: 30–300 mg/g	38.6%
Italy, 1996 [41]	1574	Night urine UAC: 20–200 *μ*g/min	32.1%
Tunisia, 2014 [44]	120	—	27.5%
Tunisia, 2008 [29]	141	24-hour urinary UAC: 30–300 mg/d	25.5%
Morocco, 2009 [13]	728	24-hour urinary UAC: 30–300 mg/d	11.5%

UAC: urinary albumin concentration.

UACR: urinary albumin creatinine ratio.

**Table 4:** Micral-test's validity in literature.

Study	Number of patients	Sensibility (%)	Specificity (%)	NPV (%)	Area under curve
Our study	**182**	**77**	**46**	**88**	**0.72**
De Grauw et al. [35]	401	67	93		0.84
Incerti et al. [36]	278	90	46		0.84
Mogensen et al. [37]	2228	96.7	71		—
Parikh et al. [38]	444	88	80		—
Sarafidis et al. [39]	165	70	83		—
Cortés-Sanabria et al. [9]	71	83	96	88	0.91
Larijani et al. [32]	200	93	87	92	
Le Floch et al. [33]	302	79	81	95	—
Mogensen et al. [37]	530	78	77		

NPV: negative predictive value.

**Table 5:** Comparison of commonly used dipsticks for the detection of albuminuria.

	Micral-test	Multistix 10 SG, Bayer [45]
Type	Semiquantitatives	Qualitative
Level of detection	20 mg/L	200 mg/L
Level of 100% specificity	35 mg/L	—
Negative predictive value	88% (20 mg/L)	73.7% (200 mg/L)
Time for reading	1 mn	1 mn
Cost	48.1£	8.59£
Efficacy of ACE treatment at screening	Reversible stage of DN	Not reversible stage of DN

ACE: angiotensin-converting enzyme (ACE) inhibitors; DN: diabetic nephropathy.
